# Successful Treatment of Scar Pain with Scrambler Therapy

**DOI:** 10.7759/cureus.5903

**Published:** 2019-10-14

**Authors:** Mark Yarchoan, Jarushka Naidoo, Thomas J Smith

**Affiliations:** 1 Oncology, Gastrointestinal Cancer, Sidney Kimmel Comprehensive Cancer Center, Johns Hopkins Medicine, Baltimore, USA; 2 Oncology, Upper Aerodigestive Cancer Program, Sidney Kimmel Comprehensive Cancer Center, Johns Hopkins Medicine, Baltimore, USA; 3 Oncology, Hospice and Palliative Medicine, Sidney Kimmel Comprehensive Cancer Center, Johns Hopkins Medicine, Baltimore, USA

**Keywords:** post-thoracotomy pain syndrome, scar pain, scrambler therapy, neuromodulation

## Abstract

Scar pain from thoracotomy, hepatectomy, or similar operations is distressing and difficult to treat. Scrambler Therapy is a novel form of superficial neuromodulation that has been effective in treating different types of neuropathic pain. We report here two cases of dramatic relief from disabling scar pain with one or two sessions of Scrambler Therapy.

## Introduction

Persistent scar pain associated with healed surgical incisions is a common and potentially debilitating type of neuropathic pain. At present, there is no universally effective treatment for persistent surgical scar pain [1]. Often-used remedies such as topical lidocaine have been shown to be no better than placebo [2-4]. Herein we describe the successful treatment of debilitating scar pain with Scrambler Therapy, which is a non-invasive form of superficial neuromodulation.

## Case presentation

Case 1

**Figure 1:**
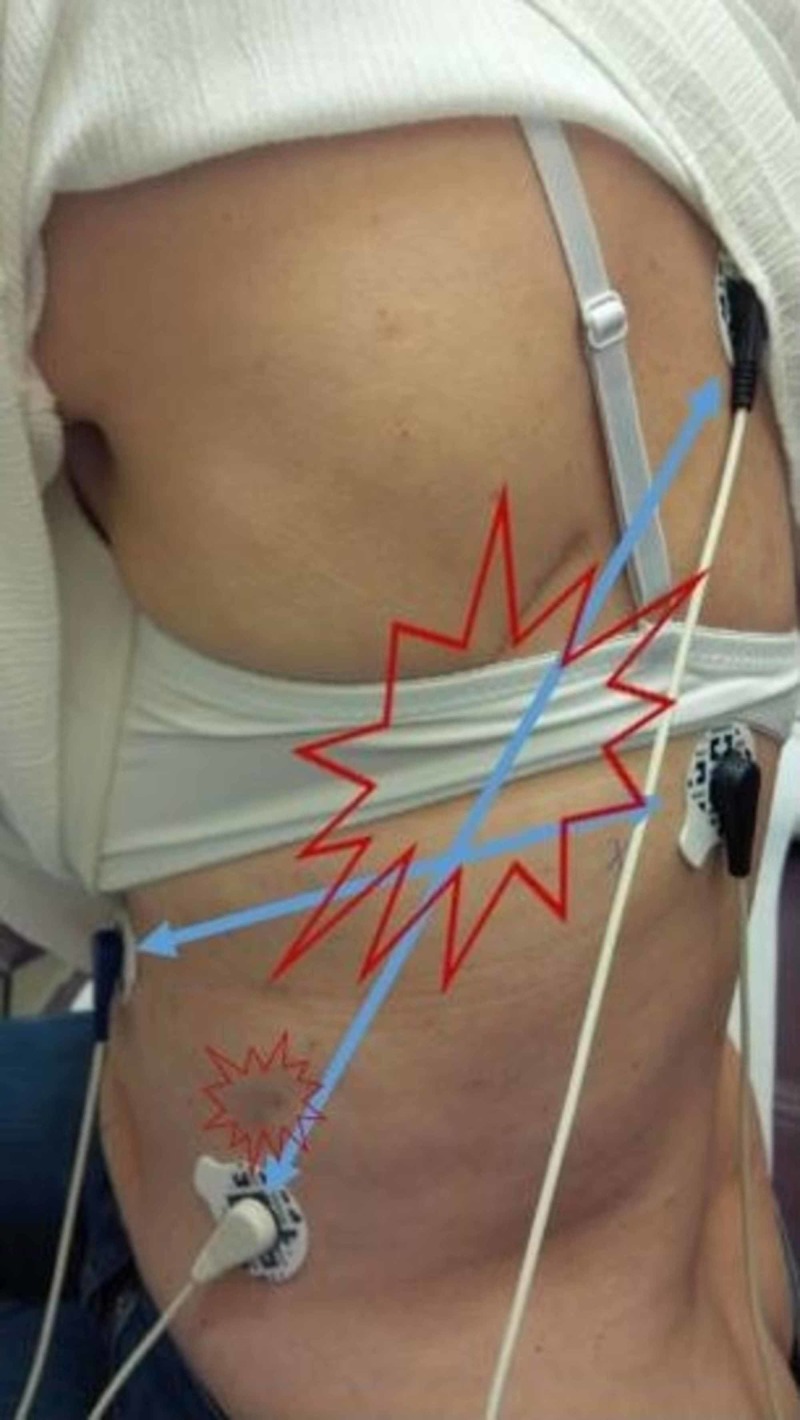
Thoracotomy scar pain The electrode pairs, shown with connecting blue lines, were positioned to capture the areas of most pain. The Scrambler Therapy signal, with an alternating current of less than 0.00029 w/cm2, travels across that area.

A 57-year-old woman underwent thoracotomy in May 2016 followed by combined chemoradiation for the treatment of a stage IIIA programmed death-ligand 1 (PD-L1) negative lung adenocarcinoma (pT3N2M0) without actionable oncogenic driver mutations. Approximately one month post-thoracotomy, she developed severe pain in the area of the thoracotomy scar and chest tube insertion site and developed biopsy-proven metastatic disease to the adrenal gland six months later. When seen in the palliative care clinic in May 2017, her clinical examination was consistent with a severe unrelenting allodynia and underlying pain rated as 9/10 that followed the distribution of the scars. With one Scrambler Therapy session, her allodynia resolved completely, and her pain was reduced to zero within 20 minutes of the 30-minute session (Figure 1). The relief lasted until November 2016, for almost six months. The patient was treated with a novel immunotherapy combination as part of a clinical trial from March 2017 until November 2017 followed by one line of systemic chemotherapy, but subsequently she died of progressive cancer.

Case 2

**Figure 2:**
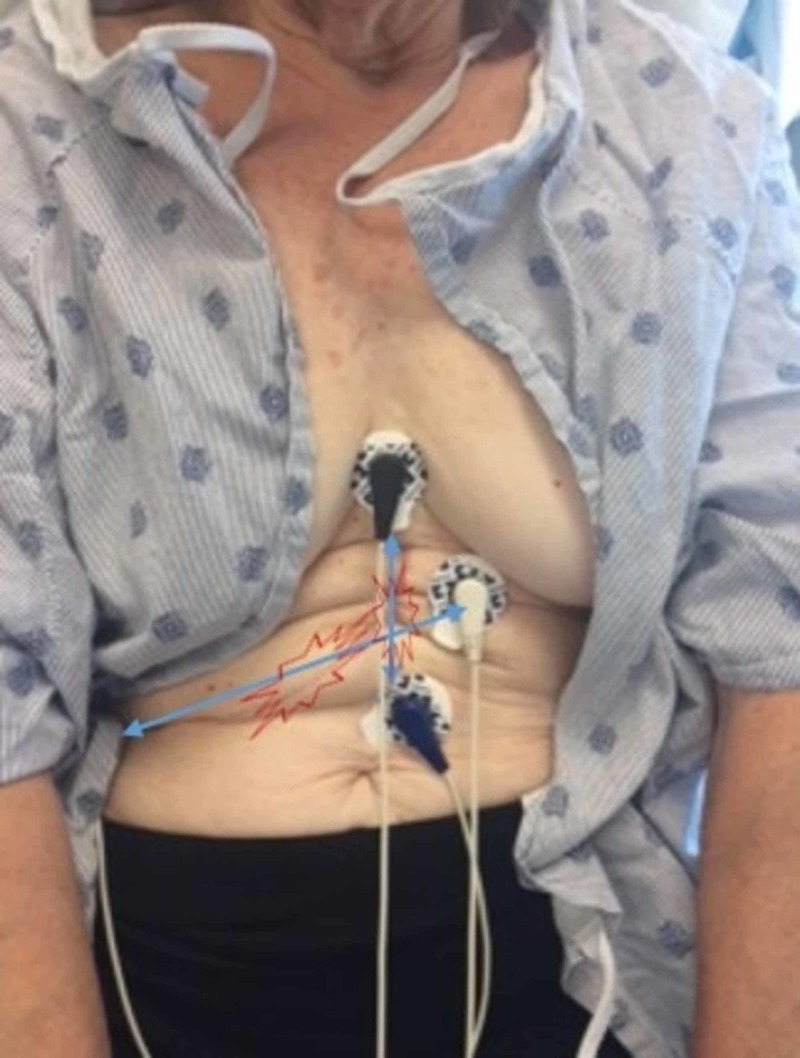
Hepatectomy scar The two long scars were treated with a set of electrodes at each end.

A 70-year-old woman underwent a partial hepatectomy for hepatocellular cancer in December 2018. She had been previously treated with neoadjuvant cabozantinib plus nivolumab on a clinical trial. About one month after surgery, she developed excruciating pain in the area of her surgical scar that prevented her from wearing a seat belt or tight-fitting clothing including a bra. She described it as a deep searing pain vertically and along the scar that went diagonally, worst 10/10, usual 2-4, 2 at rest, and 4 or greater with any movement. The examination confirmed significant allodynia, hyperalgesia, and severe pain. She was treated with one 40-minute Scrambler Therapy session with complete resolution of allodynia within 10 minutes and complete resolution of pain to zero. The allodynia relief persisted, but the pain came back after three to four days. She was treated again on day 8, with complete resolution of the pain, which has persisted (Figure 2). A few days after this second treatment, she began using topical 2% menthol [5] as directed by her palliative care physician. Three months later, the pain has never been more than 2/10.

Case reports of three or fewer patients do not require Johns Hopkins Institutional Review Board approval. The first patient is now deceased, but she previously gave verbal permission for her photograph and story to be used for teaching. The second patient gave written approval for her story and photograph to be used for teaching and publication.

## Discussion

Painful scars reduce the quality of life and alter lifestyles for patients with cancer, and there is no universally effective treatment. In both the cases, Scrambler Therapy provided quick and effective relief from allodynia and pain for months after just one or two sessions. This is similar to the effect seen with Scrambler Therapy for other superficial pains such as post-herpetic pain [6-7], with deeper tissue pain such as post-mastectomy pain [8] and complex regional pain syndrome [9], and neuropathic pain in general [10]. To date, we have not had any treatment failures in four sequentially treated patients. Further studies to ascertain the magnitude of effect in a larger population and at multiple sites are indicated.

Scrambler Therapy is a non-invasive form of electro-analgesia neuromodulation that uses five artificial neurons, sets of electrodes that send the ST signal along the affected dermatome [11]. It attempts to send synthetized “non-pain” information that looks like depolarization currents through the C-fibers to the relevant centers in the brain, without causing numbness or parasthesia. Traditional transcutaneous electrical nerve stimulation (TENS) stimulates a different set of fibers, the A-beta fibers, to block the conduction of pain impulses and causes parasthesias in the treated site. Whatever effect TENS has dissipates on the removal of the device, whereas after sufficient treatment to render the pain score to zero, Scrambler Therapy effect can last for months or longer [12]. Scrambler Therapy was designed to capture the C-fibers and relieve neuropathic pain, but its benefits have been seen in both numbness and tingling, which are mediated by different nerve fibers [13]. Because scar pain is so superficial, it may become an excellent target for such types of neuromodulation.

## Conclusions

Severe scar pain is individually uncommon but collectively common and is highly distressing without uniformly effective treatments. Scrambler Therapy gave quick and sustained relief for months with just one or two sessions, and should be studied prospectively in larger multi-site trials.
